# Machine-Learning Parsimonious Prediction Model for Diagnostic Screening of Severe Hematological Adverse Events in Cancer Patients Treated with PD-1/PD-L1 Inhibitors: Retrospective Observational Study by Using the Common Data Model

**DOI:** 10.3390/diagnostics15020226

**Published:** 2025-01-20

**Authors:** Seok Jun Park, Seungwon Yang, Suhyun Lee, Sung Hwan Joo, Taemin Park, Dong Hyun Kim, Hyeonji Kim, Soyun Park, Jung-Tae Kim, Won Gun Kwack, Sung Wook Kang, Yun-Kyoung Song, Jae Myung Cha, Sang Youl Rhee, Eun Kyoung Chung

**Affiliations:** 1 Department of Regulatory Science, College of Pharmacy, Graduate School, Kyung Hee University, Seoul 02447, Republic of Korea; psjqkr0824@naver.com (S.J.P.); syang345@khu.ac.kr (S.Y.); shsonic95@khu.ac.kr (S.H.J.); ptyoon1@gmail.com (T.P.); waterlion3@khu.ac.kr (D.H.K.); u00u96@khu.ac.kr (H.K.); 1223psy@khu.ac.kr (S.P.); 2 Institute of Regulatory Innovation Through Science (IRIS), Kyung Hee University, Seoul 02447, Republic of Korea; 3 Department of Pharmacy, College of Pharmacy, Kyung Hee University, Seoul 02447, Republic of Korea; sh198410@khu.ac.kr; 4 Department of Pharmacy, Kyung Hee University Hospital at Gangdong, Seoul 05278, Republic of Korea; jtkim@khnmc.or.kr; 5 Division of Pulmonary, Allergy and Critical Care Medicine, Kyung Hee University Hospital, Seoul 02447, Republic of Korea; wongunnim@naver.com; 6 Department of Pulmonary and Critical Care Medicine, Kyung Hee University Hospital at Gangdong, Seoul 05278, Republic of Korea; aikra@khnmc.or.kr; 7 College of Pharmacy, The Catholic University of Korea-Sungsim Campus, Bucheon 14662, Gyeonggi-do, Republic of Korea; yksong@catholic.ac.kr; 8 Division of Gastroenterology, Department of Internal Medicine, Kyung Hee University Hospital at Gangdong, Kyung Hee University School of Medicine, Seoul 05278, Republic of Korea; 9 Center for Digital Health, Medical Science Research Institute, College of Medicine, Kyung Hee University, Seoul 02447, Republic of Korea; 10 Department of Endocrinology and Metabolism, Kyung Hee University School of Medicine, Seoul 02447, Republic of Korea

**Keywords:** immune checkpoint inhibitor, PD-1 inhibitor, PD-L1 inhibitor, observational medical outcomes partnership (OMOP), common data model (CDM), real-world data (RWD), machine learning, parsimonious model, risk prediction, immune-related hematological adverse events, pharmacovigilance

## Abstract

**Background/Objectives**: Earlier detection of severe immune-related hematological adverse events (irHAEs) in cancer patients treated with a PD-1 or PD-L1 inhibitor is critical to improving treatment outcomes. The study aimed to develop a simple machine learning (ML) model for predicting irHAEs associated with PD-1/PD-L1 inhibitors. **Methods**: We utilized the Observational Medical Outcomes Partnership–Common Data Model based on electronic medical records from a tertiary (KHMC) and a secondary (KHNMC) hospital in South Korea. Severe irHAEs were defined as Grades 3–5 by the Common Terminology Criteria for Adverse Events (version 5.0). The predictive model was developed using the KHMC dataset, and then cross-validated against an independent cohort (KHNMC). The full ML models were then simplified by selecting critical features based on the feature importance values (FIVs). **Results**: Overall, 397 and 255 patients were included in the primary (KHMC) and cross-validation (KHNMC) cohort, respectively. Among the tested ML algorithms, random forest achieved the highest accuracy (area under the receiver operating characteristic curve [AUROC] 0.88 for both cohorts). Parsimonious models reduced to 50% FIVs of the full models showed comparable performance to the full models (AUROC 0.83–0.86, *p* > 0.05). The KHMC and KHNMC parsimonious models shared common predictive features including furosemide, oxygen gas, piperacillin/tazobactam, and acetylcysteine. **Conclusions:** Considering the simplicity and adequate predictive performance, our simplified ML models might be easily implemented in clinical practice with broad applicability. Our model might enhance early diagnostic screening of irHAEs induced by PD-1/PD-L1 inhibitors, contributing to minimizing the risk of severe irHAEs and improving the effectiveness of cancer immunotherapy.

## 1. Introduction

Immune checkpoint inhibitors are global breakthrough anticancer agents for the treatment of many intractable cancers including metastatic melanoma, lung cancer, renal cell carcinoma, and triple-negative breast cancer [1]. According to the pivotal clinical trial MDX010-20, commonly known as study 020, ipilimumab demonstrated survival benefits in patients with metastatic melanoma, leading to its approval as the first immune checkpoint inhibitor since 2011 [2,3,4]. The therapeutic benefits of immune checkpoint inhibitors are primarily mediated by blocking physiologic regulators to limit excessive immune responses within the body, resulting in restored T-cell immunity of the patient against cancer cells [5]. The distinct mechanisms of immune checkpoint inhibitors enhancing host immune activation are associated with immune-related adverse events (irAEs), possibly affecting all organ systems including the hematological system [5]; frequently reported immune-related hematological adverse events (irHAEs) include thrombocytopenia, leukopenia, neutropenia, and anemia, all with varying degrees of severity [6,7]. According to previous studies, the risk of irAEs was higher with cytotoxic T-lymphocyte-associated antigen 4 (CTLA-4) inhibitors compared to programmed cell death 1 (PD-1) or programmed cell death ligand 1 (PD-L1) inhibitors (overall incidence: 72% vs. 27%) [5,8,9]. Therefore, practice guidelines of clinical oncology recommend a PD-1 or PD-L1 inhibitor as the preferred first-line cancer immunotherapy to CTLA-4 inhibitors.

Despite the well-known benefits of PD-1 and PD-L1 inhibitors, early diagnosis of irAEs remains a critical challenge in oncology practice because clinical presentations of irAEs may appear similar to cancer progression or exacerbation of underlying comorbid conditions often present in patients with advanced cancer [7,10]. Furthermore, many irAEs, particularly irHAEs, might be confounded by toxicities associated with other concurrent medications such as traditional chemotherapeutic agents in patients with cancer [10]. Among irAEs, irHAEs are one of the most serious adverse events of cancer immunotherapy including PD-1 and PD-L1 inhibitors, but relatively rare, leading to difficulty in early detection for prevention of life-threatening complications [7]. Previous studies suggested the incidence of irHAEs associated with cancer immunotherapy ranging from 0.04% to 3.6% with an estimated mortality rate of 14% [11,12,13,14]. However, the risk of irAEs, including irHAEs, associated with immunotherapy might be underestimated because some irAEs were considered uncorrelated with cancer immunotherapy. Therefore, it is important to evaluate all irAEs thoroughly to provide a more comprehensive assessment of adverse events associated with anti-PD-1 or PD-L1 treatment. Once irHAEs occur, treatment with a PD-1 or PD-L1 inhibitor may need to be interrupted, possibly compromising their real-world effectiveness [7,15]. In addition, some patients who experience irHAEs may have persistent complications such as prolonged agranulocytosis, potentially leading to poorer outcomes [7,10]. Treatment with a PD-1 or PD-L1 inhibitor may be continued with close monitoring in patients with mild irAEs including hematological toxicities [16]; thus, early detection and prompt management for minimal interruption of cancer immunotherapy is critical to maintain the clinical benefits of PD-1 and PD-L1 inhibitors in real-world settings.

As aforementioned, the estimated risk for irHAEs associated with PD-1 and PD-L1 inhibitors was suggested to be highly variable [11,12,13,14]; this might be accounted for by a relative paucity of comprehensive, real-world evidence regarding the safety of immune checkpoint inhibitors. Considering substantial variability in demographic, clinical, and sociocultural factors associated with prescribing and administering medications, real-world data (RWD) in specific countries and/or ethnic groups should be assessed for safe and effective drug therapy in clinical practice [17]. In Korea, the most commonly used RWD source is the National Health Insurance (NHI) claims data [18]. However, the NHI claims data may not be the best RWD source to describe real-world experiences of PD-1 and PD-L1 inhibitors because they had been initially prescribed without insurance coverage in Korea until 2017 [19]. Furthermore, the NHI claims data do not contain imperative clinical laboratory test results to pertinently assess irHAEs associated with cancer immunotherapy [20]. Consequently, electronic medical records (EMRs) are considered a more appropriate RWD source to evaluate the real-world outcomes of treatment with a PD-1 or PD-L1 inhibitor, particularly if objective laboratory measurements are required. However, substantial time and effort are spent for securely reviewing EMRs, especially when data for numerous variables over a prolonged period of time need to be collected from multiple institutions [21]. Common data models (CDMs) such as the Observational Medical Outcomes Partnership–Common Data Model (OMOP-CDM) have emerged to provide efficient and secure access to EMR data by transformation into a standardized, anonymized data format [22].

Recent advances in machine learning (ML) algorithms for clinical risk stratification have enhanced real-time diagnostic vigilance by analyzing large-scale, multi-dimensional RWD such as multi-institutional EMRs [23,24]. Risk models can efficiently identify patients at high risk for developing irHAEs, potentially assisting clinicians in providing prompt interventions such as granulocyte colony-stimulating factor (G-CSF) or transfusions, as well as performing more targeted diagnostic tests including more frequent complete blood cell count (CBC) checks or bone marrow evaluations [15,16]. However, one of the challenges associated with ML-based risk prediction models is their complexity, leading to the models not being readily interpretable or applicable [24]. Furthermore, depending on the RWD sources (e.g., EMRs from different institutions vs. claims data), features and predictive performance of the models might be substantially variable, potentially decreasing the generalizability of the model [25]. Considering the relative lack of a simple, robust, and clinically useful prediction model for irHAEs associated with a PD-1 or PD-L1 inhibitor, the objectives of this study were to develop an ML-based parsimonious prediction model for irHAEs in patients treated with a PD-1 or PD-L1 inhibitor using the EMR-based CDM data over 6 years and to explore the generalizability of the prediction model through cross-comparison using two independent patient cohorts. To achieve the study objectives, we designed an observational study based on RWD as suggested by regulatory agencies [26]. Among a number of observational study designs such as cross-sectional, cohort, and case-control studies, case-control study design was employed to account for the potentially low incidence of irHAEs and changes in confounding variables over time within individuals, as well as to explore the risk factors of irHAEs as features in the prediction model.

## 2. Materials and Methods

### 2.1. Data Collection Source

The primary database to construct a prediction model for irHAEs was an EMR-based clinical anonymized data of Kyung Hee Medical Center (KHMC; Seoul, Republic of Korea) extracted, transformed, and loaded by the OMOP-CDM (version 5.3.1). The OMOP-CDM of KHMC, a tertiary, acute-care, university hospital, contained > 210 million medical records from 1,301,539 patients. In this study, the OMOP-CDM database of KHMC was reviewed for the period of January 2017 to January 2023 to identify eligible records for developing ML-based prediction models of irHAEs.

For exploratory cross-comparison of the primary prediction model, the OMOP-CDM database of Gangdong Kyung Hee University Medical Center (KHNMC; Seoul, Republic of Korea) was used and reviewed for the same period of time as that of KHMC (i.e., January 2017 to January 2023) to independently construct the ML-based prediction model of irHAE risk. The anonymized OMOP-CDM of KHNMC, a secondary community hospital affiliated with Kyung Hee University, contained > 250 million medical records from 920,281 patients. This study was approved by the institutional review board at Kyung Hee University (IRB No. KHSIRB-23-312[EA]), and written informed consents were exempted by the board.

### 2.2. Study Population Selection with Inclusion and Exclusion Criteria

**Figure 1:**
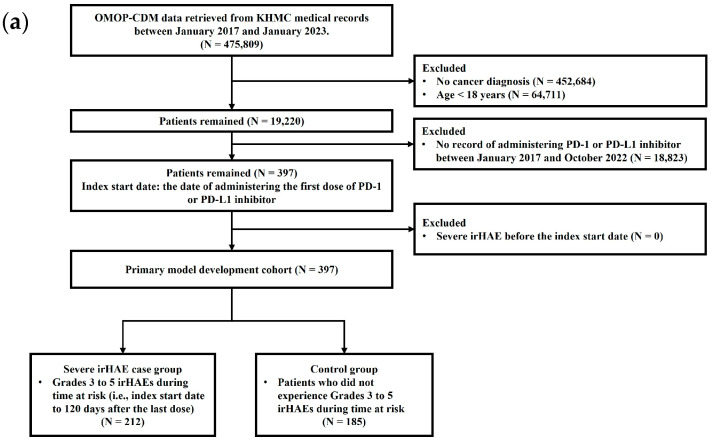

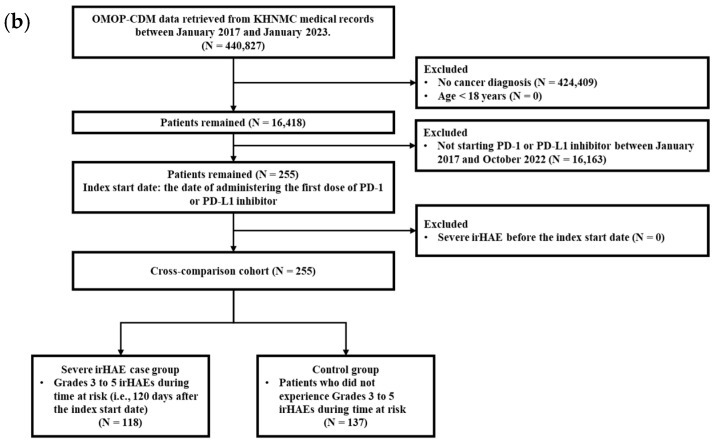
Flowchart of the study population selection: (**a**) Primary model development cohort selection (i.e., KHMC). (**b**) Cross-comparison cohort selection (i.e., KHNMC). Abbreviations: irHAE, immune-related hematological adverse event; KHMC, Kyung Hee Medical Center; KHNMC, Gangdong Kyung Hee University Medical Center; OMOP-CDM, Observational Medical Outcomes Partnership–Common Data Model; PD-1 inhibitor, programmed cell death 1 inhibitor; PD-L1 inhibitor, programmed cell death ligand 1 inhibitor.

Included patients were those at the age of 18 years or older diagnosed with any type of cancer and received at least one dose of PD-1 inhibitors (i.e., nivolumab, pembrolizumab) or a PD-L1 inhibitor (i.e., atezolizumab) from January 2017 to October 2022 (Figure 1). The index start date was the date of administering the first dose of PD-1 or PD-L1 inhibitor. The follow-up period was from the index start date to 120 days after administering the last dose of PD-1 or PD-1 inhibitor within the study period. Cohort exit date was the date of severe irHAE occurrence or study termination (i.e., 31 January 2023), whichever came first. Excluded patients were those who experienced at least one episode of severe irHAE before the index start date or those without any data records over 120 days after the last dose of PD-1 or PD-L1 inhibitor.

### 2.3. Outcome Definition

The Common Terminology Criteria for Adverse Events version 5.0 was used to define severe irHAEs including anemia, thrombocytopenia, leukopenia, and neutropenia as Grades 3 to 5 [7,27]. Briefly, anemia was defined as the blood hemoglobin concentrations < 8.0 g/dL or when transfusion or other urgent intervention was indicated. Thrombocytopenia was defined as the platelet counts in blood < 50,000/mm^3^ or when transfusion or other urgent intervention was indicated, and leukopenia as the white blood cell (WBC) counts in blood < 2000/mm^3^, and neutropenia as the neutrophil counts in blood < 1000/mm^3^.

Eligible patients were classified into two groups: case vs. control group (Figure 1). Cases were defined as patients who experienced at least one episode of severe irHAEs after initiation of PD-1 or PD-L1 inhibitor over the follow-up period. Patients with life-threatening or fatal consequences associated with severe irHAEs were also included in the case group [7,27]. Controls were those receiving a PD-1 or PD-L1 inhibitor without experiencing severe irHAEs over the follow-up period. Baseline characteristics were extracted at the index start date, including age, sex, Charlson Comorbidity Index (CCI) score, comorbid diseases based on documented diagnostic codes, and concomitant medications.

### 2.4. Analytical Procedures

#### 2.4.1. Analytical Instruments and Tools

All statistical analyses were performed using R (version 4.2.1, R Core Team 2022) as well as ATLAS (version 2.7.6), a web-based interactive application for OMOP-CDM developed by the Observational Health Data Sciences and Informatics (OHDSI) Collaborative for risk estimation and prediction as well as cohort definition. Continuous variables were statistically summarized as mean ± standard deviation (SD), and categorical variables as counts with proportions (%). Baseline characteristics of our study patients were compared statistically between the primary model development cohort (i.e., KHMC OMOP CDM database) and the cross-comparison cohort (i.e., KHNMC OMOP CDM cohort). Continuous variables were tested for normality assumption using the Shapiro–Wilk test. For normally distributed continuous variables, an independent samples t-test was conducted for comparison between the two cohorts; for those with normality assumption violated, the Wilcoxon rank-sum test (i.e., Mann–Whitney U test) was used for statistical comparison between the KHMC and KHNMC cohorts. Categorical variables were statistically compared between the two patient cohorts using a χ^2^ or Fisher exact test. Statistical significance was defined as *p* < 0.05.

#### 2.4.2. Full Model Development

Prediction models for irHAEs based on ML algorithms were developed using the “Patient-Level Prediction” (PLP) package available in ATLAS. In this study, ATLAS version 2.7.6 was used with the PLP package (version 6.3.5) imported into R (version 4.2.1). All of the following eight ML models, available in the PLP package of ATLAS, were tested: random forest, lasso logistic regression, Ada boost, decision tree, naïve Bayes, multilayer perception model, k-nearest neighbor, and gradient boost machine. The primary ML-based prediction model was developed using the KHMC CDM dataset. The cross-comparison ML prediction model was independently trained and tested using the KHNMC CDM dataset.

Throughout the ML-based model-building process, the target study population was split into the 80% training dataset and 20% test dataset with 10-fold cross-validation. Features for ML models included age, sex, medical conditions (e.g., diabetes mellitus, hypertension, etc.), clinical measurements (e.g., CCI, CHA2DS2VASc score, etc.), procedures (e.g., oxygen supply, endoscopy, etc.), and concurrent drug exposures. To ensure the inclusion of covariates documented before the occurrence of the outcomes of interest in the prediction model, filters such as “useDrugEraAnyTimePrior = TRUE” were applied as a part of the covariate setting function. Patient demographics were extracted as features on the index start date. Medical conditions were extracted over the observation period from one year before the cohort entry to the cohort exit date to include medical history as a feature. Other variables were extracted as features from the index start date to the cohort exit date.

Hyperparameters for ML models were optimized through grid-search in the PLP package without limiting the resolution. Performance of the developed ML prediction models was assessed based on the F1 score for precision and recall as well as the area under the receiver operating characteristic curve (AUROC) for accuracy. Models with AUROC ≥ 0.9 or <0.5 were not considered optimal due to overfitting and underfitting, respectively. Models with an F1 score of 0.7 or greater were suggested to have adequate classification performance.

#### 2.4.3. Model Simplification

The developed full ML prediction models were simplified to parsimonious ML (pML) models by eliminating less significant features. Significance of each feature was evaluated based on the feature importance value (FIV). Nested pML models were constructed through stepwise elimination of features from the full ML models based on FIVs; the feature with the lowest FIV was eliminated first followed by that with the next lowest FIV. The feature elimination process was repeated to construct pML models until removing all of the features accounting for the lower 25%, 40%, and 50% of the FIVs of the full ML model [28]. The optimal pML model was defined to have the fewest features among the pML models with AUROC ≥ 0.5 and <0.9, F1 score ≥ 0.7, and statistically comparable AUROC to the full ML model. The primary full ML model was simplified based on the KHMC CDM dataset. The KHNMC CDM dataset was used to independently develop the cross-comparison pML prediction model from the full cross-comparison model.

#### 2.4.4. Statistical Comparison of Full and Parsimonious Models

The performance of full and parsimonious ML models was compared based on the AUROCs using the DeLong test. When the *p*-value from the DeLong test was >0.05, the two tested models were considered to have comparable predictive performance.

## 3. Results

### 3.1. Study Population Selection

Overall, EMR data for 475,809 and 440,827 patients were available in the OMOP-CDM database of KHMC and KHNMC, respectively, over the study period. The primary KHMC cohort for model development included 19,220 adult patients diagnosed with cancer. Among them, only 397 patients received the first documented dose of a PD-1 or PD-L1 inhibitor after the diagnosis of cancer; no patients were excluded due to a prior history of severe irHAEs before the first dose of a PD-1 or PD-L1 inhibitor [Figure 1a].

The independent KHNMC cohort for exploratory cross-validation of the model included 16,418 adult patients diagnosed with cancer. Among them, only 255 patients received the first documented dose of a PD-1 or PD-L1 inhibitor after the diagnosis of cancer; no patients were excluded due to a previous history of severe irHAEs before the index start date [Figure 1b]. Figure 1 represents the detailed flowchart of the study population selection.

### 3.2. Characteristics of Study Population

Baseline characteristics of the overall study population are summarized in Table 1. Compared to patients in the cross-comparison cohort (i.e., KHNMC), those in the primary model development cohort (i.e., KHMC) had significantly more comorbid diseases, particularly diabetes mellitus and hypertension, with higher CCI and CHA2DS2VASc score, more frequently receiving concomitant chemotherapy. Otherwise, no significant differences were noted for the majority of baseline characteristics between the two cohorts (*p* > 0.05). The average age of our study patients was approximately 68 years, mostly being male (60.2% to 79.7%) treated with pembrolizumab (46.5% to 59.9%) (Table 1).

### 3.3. Characterization of Cases with irHAE Outcomes

In the primary KHMC cohort for model development (n = 397), 212 (53.4%) patients experienced at least one episode of severe irHAE after initiating a PD-1 or PD-L1 inhibitor, classified as the case group. Table 2 represents the baseline characteristics of cases in the primary model development cohort. Anemia and thrombocytopenia occurred in 171 (43.1%) and 78 (19.6%) patients, respectively. Leukopenia occurred in 98 (24.7%) patients, all presenting with neutropenia. At least two different types of irHAEs occurred simultaneously in 135 (34.0%) patients of the case group. Patients who experienced leukopenia and neutropenia more frequently received concurrent therapy with cimetidine, dexamethasone, filgrastim, or metoclopramide compared to those who experienced other irHAEs. Otherwise, the majority of baseline characteristics were comparable among the irHAE subgroups (i.e., anemia, thrombocytopenia, and leukopenia/neutropenia) (Table 2). The control group included the remaining 185 patients (46.6%) who did not experience any episode of severe irHAE documented in the OMOP-CDM database [Figure 1a].

For exploratory cross-comparison, 118 (46.3%) patients experienced at least one episode of severe irHAE after initiating a PD-1 or PD-L1 inhibitor, defining them as the case group; the remaining 137 (53.7%) patients were in the control group [Figure 1b]. Table 3 shows the baseline characteristics of patients in the case group of the cross-comparison cohort. Anemia and thrombocytopenia occurred in 1 (0.8%) and 84 (71.2%) patients, respectively. A total of 83 (70.3%) patients experienced leukopenia, all simultaneously presenting with neutropenia. At least two different types of irHAEs occurred concurrently in 50 (42.4%) patients of the case group. Patients who experienced leukopenia and neutropenia were more frequently co-administered megestrol compared to those who experienced thrombocytopenia. Otherwise, the majority of baseline characteristics were comparable among the irHAE subgroups (Table 3).

### 3.4. Development of Full Prediction Model

Table 4 summarizes the model performance of the tested ML algorithms using the primary model development cohort and the cross-comparison cohort. Gradient boost machine and k-nearest neighbor algorithms failed to converge; therefore, no full model was constructed with these two algorithms. Among the tested ML algorithms, random forest showed the best predictive performance with a low risk of overfitting for both the primary model development cohort (i.e., KHMC) and the cross-comparison cohort (i.e., KHNMC), resulting in comparable predictive performance between the primary KHMC and the cross-comparison KHNMC models (KHMC: AUROC 0.88, F1 score 0.8; KHNMC: AUROC 0.88, F1 score 0.7) (Table 4). Hyperparameters for the best full prediction model based on random forest were optimized as follows: max depth (i.e., maximal number of interactions) of 10, mtries (i.e., the number of features included in each tree) of the square root of the total number of features, and ntrees (i.e., the number of trees built) of 25,000. For both the model development and cross-comparison cohorts, an extensive set consisting of 2438 features such as demographics, medications, concomitant diseases, and medical procedures was initially considered. The number of features included in the best full model was 449 for the primary KHMC model and 478 for the cross-comparison KHNMC model.

### 3.5. Simplification of Full Prediction Models

**Figure 2:**
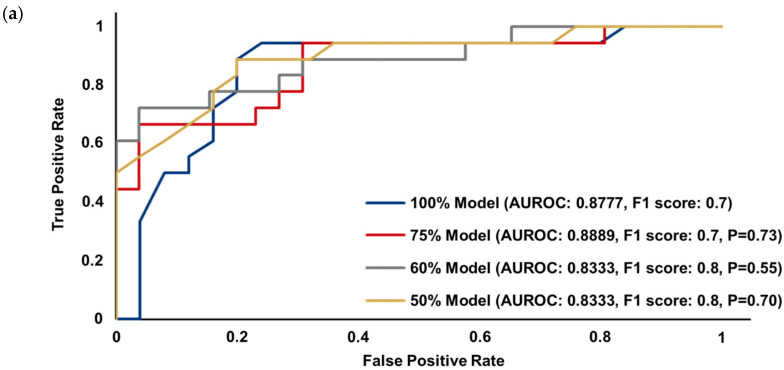

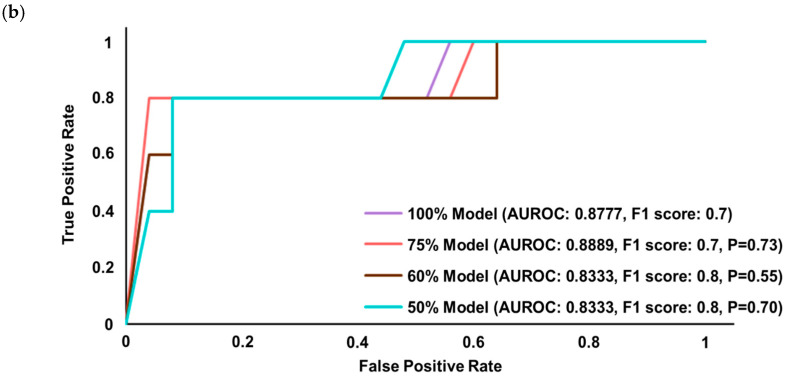
Comparative predictive performance of the parsimonious prediction models based on random forest as well as the full prediction model for (**a**) the primary model development cohort (i.e., KHMC) and (**b**) the cross-comparison cohort (i.e., KHNMC). Abbreviations: 100% Model, the full prediction model; 75% Model, the parsimonious model with reduced features with the sum of their importance values to be 75% of the full model; 60% Model, the parsimonious model with reduced features with the sum of their importance values to be 60% of the full model; 50% Model, the parsimonious model with reduced features with the sum of their importance values to be 50% of the full model; AUROC, area under the receiver operating characteristic curve; *p*-value from the DeLong test for comparison to the 100% full model based on each cohort; KHMC: Kyung Hee Medical Center; KHNMC: Gangdong Kyung Hee University Medical Center.

Because the full prediction models were developed based on the random forest algorithm, the primary and cross-comparison full models were incrementally simplified to the corresponding pML models using the embedded method based on the FIV [29]. Figure 2 shows the predictive performance of the pML models with the sum of FIVs reduced to 75% (i.e., 75% pML model), 60% (i.e., 60% pML model), and 50% (i.e., 50% pML model) of the full models. For both the KHMC and the KHNMC cohorts, the predictive performance of pML models including 50% pML models and the full model was suggested to be comparable (AUROC of KHMC models: 0.8785 for the full model vs. 0.8731 for 75% pML model vs. 0.8692 for 60% pML model vs. 0.8623 for 50% pML model, *p* > 0.05; AUROC of KHNMC models: 0.8777 for the full model vs. 0.8889 for 75% pML model vs. 0.8333 for 60% pML model vs. 0.8333 for 50% pML model, *p* > 0.05). Among the pML models, the 50% pML models included the fewest features; therefore, with comparable predictive performance to the full models, the 50% pML model was suggested to be the optimal pML model.

### 3.6. Cross-Comparison of Simplified Prediction Models

**Figure 3:**
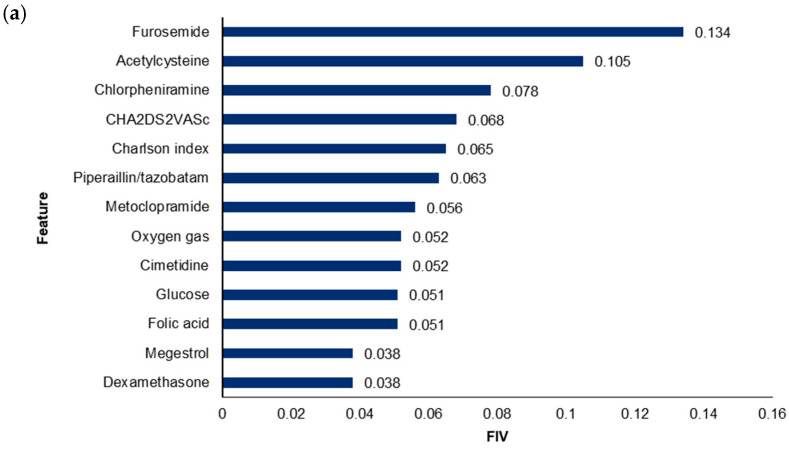

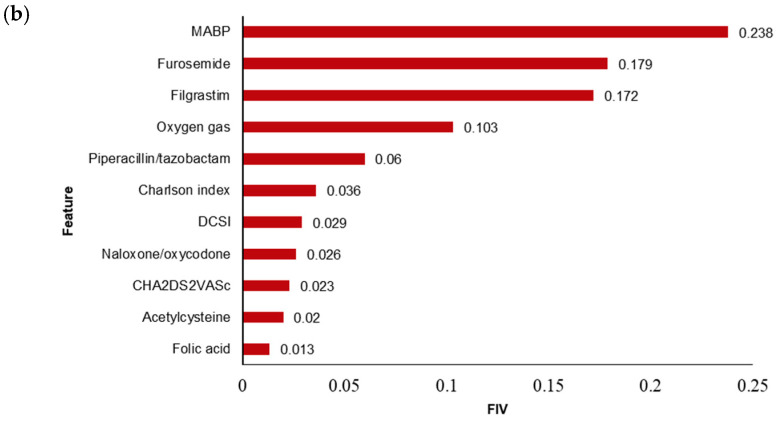
Importance of features included in the final parsimonious model for (**a**) the primary model development cohort (i.e., KHMC) and (**b**) the cross-comparison cohort (i.e., KHNMC). DCSI: diabetes complications severity index; FIV: feature importance value; MABP: mean arterial blood pressure.

The optimal pML model included 36 features for the primary KHMC model and 44 features for the cross-comparison KHNMC model. Figure 3 shows the 10 most important features based on the FIVs included in each of the primary KHMC and the cross-comparison KHNMC pML model. The primary and the cross-comparison pML models shared the following features as the most important features: concomitant treatment with furosemide (FIV = 0.134 for the KHMC model, 0.179 for the KHNMC model), acetylcysteine (FIV = 0.105 for KHMC, 0.02 for KHNMC), and piperacillin/tazobactam (FIV = 0.063 for KHMC, 0.060 for KHNMC); clinical scores of CHA2DS2VASc (FIV = 0.068 for KHMC, 0.023 for KHNMC) and CCI (FIV = 0.065 for KHMC, 0.036 for KHNMC); and concurrent administration of oxygen gas (FIV = 0.052 for KHMC, 0.103 for KHNMC).

## 4. Discussion

To our knowledge, this is one of the first few studies utilizing EMR-based OMOP-CDM data to develop an ML-based prediction model of the irHAE risk for patients with cancer treated with a PD-1 or PD-L1 inhibitor. Our study showed irHAEs commonly occur in patients with cancer treated with a PD-1 or PD-L1 inhibitor (53.4% in KHMC; 46.3% in KHNMC) (Table 1), suggesting the imperative need for a prediction model to identify patients at a higher risk for developing irHAEs. We developed a single ML model to predict the overall risk for irHAEs as a composite event including anemia, thrombocytopenia, leukopenia, and neutropenia rather than multiple models for risk prediction of individual-specific irHAEs because a substantial proportion of our study patients simultaneously experienced two or more types of irHAEs (Table 2 and Table 3). As a result, statistics as well as the ML models were presented for the composite irHAEs rather than each of the specific irHAEs. Our prediction model for the composite irHAEs might be more clinically plausible in practice because, as shown in our present study, a substantial number of real-world patients receiving a PD-1 or PD-L1 inhibitor experience multiple types of irHAEs concurrently. However, caution needs to be exercised when applying our model to predict the risk for a specific irHAE induced by a PD-1 or PD-L1 inhibitor; the majority of patients included in our prediction model had two or more types of irHAEs. Considering the risk of irHAEs potentially leading to treatment discontinuation and even death [7,10], we anticipate the RWD-based prediction model may contribute to improving diagnostic workflow and, ultimately, enhancing the clinical benefit of immunotherapy through identification of high-risk patients for irHAEs and early detection of irHAEs in patients receiving a PD-1 or PD-L1 inhibitor. In this present study, we developed a strategically streamlined ML prediction model simplified by including a select few features based on feature importance with its predictive performance comparable to the full ML prediction model (Figure 2). The prediction model developed by using the primary model development cohort was compared with the cross-comparison model based on the external cohort to explore the generalizability and promote the clinical applicability of our newly developed prediction model. Overall, we have successfully developed a reliable pML model simplified to reduce the complexity of full ML models with generalizability assessed by cross-comparison using an external dataset, enhancing the clinical utility and applicability of the ML-based screening model to predict the risk of irHAEs for patients receiving a PD-1 or PD-L1 inhibitor.

In this study, we utilized two independent patient cohorts: one for primary model development (i.e., KHMC dataset) and the other one for cross-comparison (i.e., KHNMC dataset). Compared to the KHNMC cohort, patients in the KHMC cohort had a relatively poor prognosis as suggested by higher CCI scores and a higher risk of bleeding with more comorbidities as implied by CHA2DS2VASc score, more frequently receiving concomitant chemotherapy (Table 1). This difference in the baseline characteristics might be associated with the different levels of care between the two institutions; while KHNMC is a secondary, community hospital affiliated with a university, KHMC is a tertiary, acute-care, university teaching hospital primarily providing care to patients with higher severity. Other characteristics including age and concurrent therapy were mostly similar between the two patient cohorts. In both institutions, irHAEs were more likely to occur in patients with a relatively higher CCI score, higher CHA2DS2VASc score, and more comorbid diseases more often receiving concurrent therapy (Table 1). Notably, concomitant chemotherapy was more frequently used in control groups (90.3% vs. 59.0% in KHMC, 71.5% vs. 34.7% in KHNMC), possibly due to a potentially better functional status in the control cohort compared to the cases based on the CCI score (Table 1). This is consistent with previous studies suggesting a worse performance status defined as the Eastern Cooperative Oncology Group (ECOG) score of two or higher as a risk factor for worse outcomes in patients receiving chemotherapy in combination with immune checkpoint inhibitors, discouraging the use of a PD-1 or PD-L1 inhibitor in combination with chemotherapy in patients with worse performance status [30,31,32,33]. Overall, our study population is an appropriate representative of real-world patients with cancer treated with a PD-1 or PD-L1 inhibitor [30,31,32,33], suggesting the validity of the data source to develop the prediction model of irHAEs associated with a PD-1 or PD-L1 inhibitor.

To overcome the complexity of ML-based models with numerous features, our present study developed pML models by reducing the number of features included in the full model. The pML models were developed from the full ML models by reducing the number of features in the model through the embedded method simply based on the FIV; the FIV-based embedded method appropriately selects features for the ML models based on random forest as in our current study [29]. The number of features included in the final pML models was substantially reduced to <10% of those in the full ML models (36 features reduced from 449 for the primary model; 44 from 478 for the cross-comparison model). Nonetheless, the predictive performance of the final pML models was comparable to the full ML models for both the primary and cross-comparison cohorts (Figure 2), suggesting effective prediction of the risk for severe irHAEs associated with a PD-1 or PD-L1 inhibitor without substantial computational demands (AUROC: 0.8623 for the primary model, 0.8333 for the cross-comparison model; *p* > 0.05 compared to the full model for both). Therefore, our pML prediction models primarily focusing on critical clinical features might enhance the clinical utility and applicability of ML-based prediction models to assist with decision making in clinical practice with much less complexity in the model structure requiring less computational capacity. Overall, our model development and subsequent simplification strategy might contribute to expanding the application of pML models in clinical practice for various patient populations.

In contrast to previous studies suggesting a variety of biomarkers as risk factors of irAEs associated with immune checkpoint inhibitors including a PD-1 or PD-L1 inhibitor [34,35,36,37,38,39,40,41], no biomarkers were utilized as potential features in our model. Recently, Sung and colleagues developed an ML-based prediction model for irAEs of immune checkpoint inhibitors in 672 patients with cancer using various biomarker-based features such as germline exomes (e.g., human leukocyte antigens), transcriptomes (e.g., gene expression profile, immune cell expression profile), and laboratory tests including complete blood cell count and serum chemistry (e.g., neutrophil–lymphocyte ratio) [42]. Nonetheless, the predictive performance of our model was comparable to or slightly better than the biomarker-based ML model (AUROC: 0.8333 to 0.8785 vs. 0.80) (Figure 2). Considering additional costs and procedures to obtain various biomarkers, biomarker-based ML models might be relatively inconvenient for routine use in clinical practice. The absence of biomarkers in our ML models might further improve the clinical feasibility and utility without compromising the predictive performance of the prediction models.

Through the development of separate ML-based prediction models for two independent patient cohorts (i.e., primary model development and cross-comparison cohort), common clinical features in the final pML models were identified and subsequently, suggested to be the critical factors to predict the risk for irHAEs of a PD-1 or PD-L1 inhibitor. Some of the features were exclusively included in either the KHMC or the KHNMC model; this might be accounted for by the difference in the patient population or variability in practice patterns at each institution. The majority of common critical features (Figure 3) have been previously reported to be risk factors for irAEs of immune checkpoint inhibitors [43]. Our present study is one of the first few studies specifically confirming an increased risk for irHAEs of a PD-1 or PD-L1 inhibitor in association with these risk factors. Notably, most of the common critical features were related to concomitant therapy (i.e., furosemide, acetylcysteine, piperacillin/tazobactam, and oxygen gas) (Figure 3). However, the underlying mechanisms regarding the increased risk for irHAEs owing to concomitant therapy are not clarified yet. Additional studies are warranted to confirm the causality and explain the underlying mechanisms of the increased risk for irHAEs related to concomitant therapy (e.g., drug interactions, comorbid disease requiring concurrent treatment, etc.). Overall, our pML model developed by focusing on common clinical features is a feasible, reliable, efficient, easy-to-use model in clinical practice to predict the risk for irHAEs associated with a PD-1 or PD-L1 inhibitor in patients with cancer. Our approach to simplifying the models to parsimonious versions represents a significant advancement by reducing complexity and computational demands, making it clinically applicable. The validation of our prediction models across two independent datasets (KHMC and KHNMC) has enhanced their generalizability. Furthermore, by utilizing EMR-based CDM data from real-world settings, our findings are more applicable to typical clinical environments, demonstrating the utility of RWD from the perspectives of clinical practice as well as research settings.

Our prediction models hold significant diagnostic implications for clinicians managing patients with cancer receiving a PD-1 or PD-L1 inhibitor. Model-based identification of high-risk patients and early detection of irHAEs might contribute to lowering morbidities and mortalities associated with irHAEs as well as improving the overall treatment outcomes [7,10,15]. Considering the difficulty in diagnosis of irHAEs due to numerous confounding factors such as the progression of underlying cancer and myelosuppression associated with medications other than a PD-1 or PD-L1 inhibitor, an accurate, reliable prediction model is imperative for early diagnosis and timely intervention of irHAEs [10,20]. Our ML-based prediction model based on common clinical features such as comorbid diseases, concurrent medications, and demographics, showed adequate predictive performance, suggesting that it is a clinically useful screening tool for clinicians to proactively assess the risk for irHAEs and to diagnose irHAEs in a timely manner [16]. Furthermore, the parsimonious design of our prediction model confers practical advantages in real-world diagnostic settings. With fewer features included in the pML models compared to traditional ML models, pML models may be more easily implemented in EMR systems without substantial computational demands [23,24]. Therefore, our approach could seamlessly integrate into clinical decision support systems, providing a framework for an early alert system to improve diagnostic efficiency, optimize interventions for irHAEs, and ultimately support precision management strategies for patients treated with a PD-1 or PD-L1 inhibitor [10,43].

There are study limitations to be addressed. First, the primary prediction model was developed using the CDM data at a single institution (i.e., KHMC). Although the cross-comparison model was constructed and subsequently compared with the primary prediction model to assess model reliability, direct validation of the primary prediction model was not performed using an external cohort due to the limited functionality of the ATLAS tool for CDM data analysis. In addition, our current study was conducted using CDM data exclusively from two institutions with a relatively small sample size (*n* = 652 total; *n* = 397 in KHMC; *n* = 255 in KHNMC). Consequently, our patient cohort might not contain sufficient samples to comprehensively identify predictors of the irHAE risk associated with a PD-1 or PD-L1 inhibitor. Additionally, biomarkers were not integrated into our prediction models, although our model performance was not adversely impacted without biomarkers. At the expense of increased cost and complexity, future studies might be warranted to incorporate biomarkers to potentially improve the predictive accuracy of prediction models. Furthermore, our study cohorts exclusively included Korean institutions; therefore, our findings might not be directly applicable to other ethnicities or healthcare systems in other countries without further validation. Moreover, due to the binary nature of drug exposure and laboratory measurement data in the CDM, we could not clearly assess the quantitative contribution of baseline clinical data such as complete blood cell counts as a continuous variable to the risks for irHAEs [22,44,45]. Additionally, the transformed data in the CDM based on EMRs were limited and not specific enough, making it extremely challenging to specify the type of cancer being treated. Consequently, although the particular cancer type might affect the risk for irHAEs including hemorrhage risks, the specific confirmed diagnosis of cancer types being treated could not be identified or tested as potential features in our prediction model. Thus, caution needs to be exercised when extrapolating our prediction models to general patient populations with diverse clinical characteristics and genetic compositions. Moreover, due to the retrospective observational nature of our present study based on EMR-based CDM data, our study findings might be confounded by related clinical data or missing data, making it difficult to establish causality between features and irHAE risk [22,44,45]. A single feature predictive of the risk for irHAEs might be confounded by various factors such as clinician judgment and patient preferences. Although various patient factors such as chemotherapy experiences were tested to address this limitation, differentiation of irHAEs from hematological adverse events resulting from chemotherapy or other factors (e.g., anticoagulant use, underlying disease) was extremely challenging due to our study design. Additional research might be needed such as integration of the NHI claims data to supplement missing data in the EMR-based CDM, mechanistic studies, or prospective clinical studies to assess the causal relationship in addition to associations between features and irHAE risk. Lastly, our prediction models were simplified only based on FIVs. The FIV-based method for model simplification was considered appropriate in our study because the structure of our prediction model was the tree-based random forest algorithm [29]. However, caution should be exercised for simplifying prediction models constructed by other algorithms than the tree-based approach; more complicated methods such as GA-PARSIMONY might be more universal to develop pML models with various ML algorithms [46]. Overall, multi-center international, preferably prospective, studies with a larger sample size and external validation are warranted to enhance the generalizability and clinical applicability of our prototypical prediction models in clinical practice.

In further study, our prediction models should be validated with larger, ethnically diverse populations across multiple countries to improve the generalizability of our findings. Additionally, the integration of biomarkers into our model should be explored to further enhance the model performance, particularly in environments where such biomarker testing is feasible. Furthermore, prospective clinical studies are warranted to establish causality between features and irHAE risk as well as to confirm our present study findings.

## 5. Conclusions

In conclusion, irHAEs frequently occur in patients receiving a PD-1 or PD-L1 inhibitor. Using various demographic and clinical characteristics in the EMR-based CDM as features, the ML-based prediction model was successfully developed with adequate predictive performance; the prediction model based on random forest performed best at predicting the risk for irHAEs in patients treated with a PD-1 or PD-L1 inhibitor. To enhance the clinical utility and applicability of the ML-based prediction models in clinical practice, the full model was successfully simplified to the pML model to contain only a few select critical features based on FIVs without compromising the predictive performance. Our pML prediction model demonstrated significant diagnostic utility with readily available clinical information from EMR-based CDM data as model features, rapidly identifying high-risk patients and timely diagnostic and therapeutic interventions for irHAEs. Nonetheless, we acknowledge the need for improved generalizability through broader validation, particularly in global, ethnically diverse population settings, to confirm the findings of this present study and establish a clear causal relationship between patient-related factors and the risk for irHAEs. Future studies with a larger sample size and external validation may expand the applicability of our prediction models and apply our modeling methodology to other therapeutic areas.

## Figures and Tables

**Table 1:** Baseline characteristics of the overall study population.

	Primary Model Development Cohort ^a^		Cross-Comparison Cohort ^b^	
	Cases (N = 212)	Controls (N = 185)	Cases (N = 118)	Controls (N = 137)
**Age (years) ^c^**				
Mean ± SD	67.8 ± 10.3	68.4 ± 11.3	66.2 ± 9.8	67.8 ± 10.6
**Gender ^d^, N (%)**				
Male	169 (79.7)	140 (75.7)	71 (60.2)	98 (71.5)
Female	43 (20.3)	45 (24.3)	47 (39.8)	39 (28.5)
**Type of cancer being treated, N (%)**				
Solid organ	161 (77.8)	124 (67.0)	45 (38.1)	89 (65.0)
Hematological	61 (28.8)	33 (17.8)	37 (31.4)	58 (42.3)
Both	10 (4.7)	0 (0.0)	0 (0.0)	10 (7.3)
Unknown	0 (0.0)	28 (15.1)	36 (30.5)	0 (0.0)
**CCI score ^d,e^, mean ± SD**	7.4 ± 3.7	7.0 ± 3.9	6.7 ± 3.0	6.2 ± 3.2
**CHA2DS2VASc score ^d,f^, mean ± SD**	2.4 ± 1.4	2.2 ± 1.5	1.9 ± 1.2	1.7 ± 1.3
**Comorbid disease, N (%)**				
Atrial fibrillation ^c^	7 (3.3)	4 (2.2)	5 (4.2)	6 (4.4)
Chronic kidney disease ^c^	8 (3.8)	16 (8.6)	4 (3.4)	5 (3.6)
Diabetes mellitus ^d^	63 (29.7)	44 (23.8)	26 (22.0)	19 (13.9)
Hypertension ^d^	98 (46.2)	65 (35.1)	31 (26.3)	30 (21.9)
Thromboembolism ^c^	11 (5.2)	7 (3.8)	8 (6.8)	3 (2.2)
**Concomitant chemotherapy ^d^, N (%)**	125 (59.0)	167 (90.3)	41 (34.7)	98 (71.5)
**Immune checkpoint inhibitor ^c^, N (%)**				
Atezolizumab	65 (30.7)	55 (29.7)	31 (26.3)	36 (26.3)
Nivolumab	22 (10.4)	26 (14.1)	23 (19.5)	22 (16.1)
Pembrolizumab	127 (59.9)	86 (46.5)	65 (55.1)	80 (58.4)
**Concurrent therapy, N (%)**				
Acetylcysteine ^c^	76 (35.8)	31 (16.8)	32 (27.1)	23 (16.8)
Chlorpheniramine ^d^	149 (70.3)	64 (34.6)	105 (89.0)	63 (46.0)
Cimetidine ^d^	89 (42.0)	40 (21.6)	15 (12.7)	14 (10.2)
Dexamethasone ^c^	131 (61.8)	61 (33.0)	63 (53.4)	60 (43.8)
Filgrastim ^c^	24 (11.3)	0 (0.0)	9 (7.6)	2 (1.5)
Folic acid ^c^	59 (27.8)	21 (11.4)	29 (24.6)	34 (24.8)
Furosemide ^c^	98 (46.2)	47 (25.4)	69 (58.5)	33 (24.1)
Glucose ^c^	76 (35.8)	29 (15.7)	23 (19.5)	7 (5.1)
Megestrol ^c^	92 (43.4)	44 (23.8)	46 (39.0)	31 (22.6)
Metoclopramide ^c^	119 (56.1)	62 (33.5)	55 (46.6)	50 (36.5)
Naloxone/oxycodone ^c^	73 (34.4)	38 (20.5)	33 (28.0)	25 (18.2)
Oxygen gas ^c^	101 (47.6)	53 (28.6)	63 (53.4)	43 (31.4)
Piperacillin/tazobactam ^c^	91 (42.9)	33 (17.8)	46 (39.0)	24 (17.5)

^a^ Data from Kyung Hee Medical Center. ^b^ Data from Gangdong Kyung Hee University Medical Center. ^c^ *p* > 0.05 for comparison between the primary model development and the cross-comparison cohort. ^d^ *p* < 0.05 for comparison between the primary model development and the cross-comparison cohort. ^e^ CCI, Charlson comorbidity index; scores ranging from a minimum of 0 to a maximum of 28. ^f^ CHA2DS2VASc score ranging from a minimum of 0 to a maximum of 10.

**Table 2:** Baseline characteristics of irHAE cases in the primary KHMC model development cohort ^a^.

	irHAE Cases (N = 212)		
	Anemia (N = 171)	Thrombocytopenia (N = 78)	Leukopenia ^b^ (N = 98)
**Age (years)**			
Mean ± SD	68.0 ± 8.7	67.7 ± 9.2	68.5 ± 9.4
**Gender, N (%)**			
Male	130 (76.0)	64 (82.1)	82 (83.7)
Female	41 (24.0)	14 (17.9)	16 (16.3)
**Type of cancer being treated, N (%)**			
Solid organ	133 (77.8)	61 (78.2)	76 (77.6)
Hematological	50 (29.2)	22 (28.2)	28 (28.6)
Both	8 (4.7)	5 (6.4)	6 (6.1)
Unknown	0 (0.0)	0 (0.0)	0 (0.0)
**CCI score ^c^, mean ± SD**	8.5 ± 3.6	8.9 ± 3.8	7.8 ± 3.5
**CHA2DS2VASc score ^d^,** **mean ± SD**	2.4 ± 1.5	2.2 ± 1.6	2.3 ± 1.5
**Comorbid disease, N (%)**			
Atrial fibrillation	6 (3.5)	7 (9.0)	7 (7.1)
Chronic kidney disease	8 (4.7)	6 (7.7)	5 (5.1)
Diabetes mellitus	52 (30.4)	16 (20.5)	29 (29.6)
Hypertension	84 (49.1)	32 (41.0)	44 (44.9)
Thromboembolism	9 (5.3)	4 (5.1)	3 (3.1)
**Concomitant chemotherapy,** **N (%)**	101 (59.1)	46 (59.0)	58 (59.2)
**Immune checkpoint inhibitor, N (%)**			
Atezolizumab	59 (34.5)	30 (38.5)	36 (36.7)
Nivolumab	30 (17.5)	9 (11.5)	10 (10.2)
Pembrolizumab	85 (49.7)	39 (50.0)	53 (54.1)
**Concurrent therapy, N (%)**			
Acetylcysteine	52 (30.4)	28 (35.9)	29 (29.6)
Chlorpheniramine	121 (70.8)	63 (80.8)	66 (67.3)
Cimetidine	61 (35.7)	34 (43.6)	59 (60.2)
Dexamethasone	95 (55.6)	53 (68.0)	73 (74.5)
Filgrastim	19 (11.1)	9 (11.5)	11 (11.2)
Folic acid	33 (19.3)	18 (23.1)	22 (22.4)
Furosemide	75 (43.9)	41 (52.6)	43 (43.9)
Glucose	52 (30.4)	34 (43.6)	33 (33.7)
Megestrol	71 (41.5)	28 (35.9)	45 (45.9)
Metoclopramide	86 (50.3)	48 (61.5)	66 (67.3)
Naloxone/oxycodone	59 (34.5)	27 (34.6)	34 (34.7)
Oxygen gas	77 (45.0)	53 (28.6)	63 (53.4)
Piperacillin/tazobactam	69 (40.4)	38 (48.7)	36 (36.7)

Abbreviations: irHAE, immune-related hematological adverse event; KHMC, Kyung Hee Medical Center; SD, standard deviation; CCI, Charlson comorbidity index. ^a^ Data from the OMOP-CDM database of Kyung Hee Medical Center. ^b^ All simultaneously with neutropenia. ^c^ CCI scores ranging from a minimum of 0 to a maximum of 28. ^d^ CHA2DS2VASc score ranging from a minimum of 0 to a maximum of 10.

**Table 3:** Baseline characteristics of irHAE cases in the KHNMC cross-comparison cohort ^a^.

	irHAE Cases (N = 118) ^b^	
	Thrombocytopenia (N = 84)	Leukopenia ^c^ (N = 83)
**Age (years)**		
Mean ± SD	64.4 ± 9.6	64.9 ± 9.6
**Gender, N (%)**		
Male	44 (52.4)	54 (65.1)
Female	40 (47.6)	29 (34.9)
**Type of cancer being treated, N (%)**		
Solid organ	32 (38.1)	31 (37.3)
Hematological	26 (31.0)	27 (32.5)
Both	0 (0.0)	0 (0.0)
Unknown	26 (31.0)	25 (30.1)
**CCI score ^d^, mean ± SD**	7.0 ± 3.0	6.3 ± 3.2
**CHA2DS2VASc score ^e^, mean ± SD**	1.9 ± 1.2	1.6 ± 1.1
**Comorbid disease, N (%)**		
Atrial fibrillation	1 (1.2)	2 (2.4)
Chronic kidney disease	3 (3.6)	3 (3.6)
Diabetes mellitus	15 (17.9)	9 (10.8)
Hypertension	19 (22.6)	15 (18.1)
Thromboembolism	4 (4.8)	2 (2.4)
**Concomitant chemotherapy, N (%)**	29 (34.5)	29 (34.9)
**Immune checkpoint inhibitor, N (%)**		
Atezolizumab	14 (21.5)	18 (31.6)
Nivolumab	12 (18.5)	9 (15.8)
Pembrolizumab	39 (60.0)	31 (54.4)
**Concurrent therapy, N (%)**		
Acetylcysteine	21 (25.0)	14 (16.9)
Chlorpheniramine	62 (73.8)	48 (57.8)
Cimetidine	14 (16.7)	13 (15.7)
Dexamethasone	36 (42.9)	36 (43.4)
Filgrastim	2 (2.4)	7 (8.4)
Folic acid	17 (20.2)	13 (15.7)
Furosemide	43 (51.2)	29 (34.9)
Glucose	17 (20.2)	9 (10.8)
Megestrol	26 (31.0)	37 (44.6)
Metoclopramide	32 (38.1)	32 (38.6)
Naloxone/oxycodone	24 (28.6)	29 (34.9)
Oxygen gas	35 (41.7)	22 (26.5)
Piperacillin/tazobactam	26 (31.0)	23 (27.7)

Abbreviations: irHAE, immune-related hematological adverse event; KHNMC, Gangdong Kyung Hee University Medical Center; SD, standard deviation; CCI, Charlson comorbidity index. ^a^ Data from the OMOP-CDM database of Gangdong Kyung Hee University Medical Center. ^b^ Include one patient with anemia. ^c^ All simultaneously with neutropenia. ^d^ CCI scores ranging from a minimum of 0 to a maximum of 28. ^e^ CHA2DS2VASc score ranging from a minimum of 0 to a maximum of 10.

**Table 4:** Predictive performance of full prediction models using various machine learning algorithms ^a^.

Algorithms	Primary Model Development Cohort			Cross-Comparison Cohort		
	AUROC	AUPRC	F1 Score	AUROC	AUPRC	F1 Score
Lasso logistic regression	0.97	0.95	0.7	0.67	0.24	0.4
Random forest	0.88	0.83	0.8	0.88	0.60	0.7
Ada boost	0.81	0.70	0.7	0.90	0.77	0.4
Decision tree	0.63	0.51	0.6	0.79	0.53	0.5
Naïve Bayes	0.63	0.50	0.6	0.51	0.18	0.2
Multilayer perception	0.52	0.43	0.5	0.49	0.17	0.3

Abbreviations: AUROC, area under the receiver operating characteristic curve; AUPRC, area under the precision–recall curve. ^a^ Gradient boost machine and k-nearest neighbor algorithms were tested but failed to converge; no full model was constructed.

## Data Availability

The datasets generated and/or analyzed during the current study are not publicly available due to the inclusion of private medical information in the anonymized OMOP CDM data. Therefore, access to the OMOP CDM data of our institution is restricted to internal private networks; however, data may be available from the corresponding authors on reasonable request.
